# Supplementary material for: Modeling glioblastoma heterogeneity as a dynamic network of cell states
Source: Mol Syst Biol. 2021 Sep 16;17(9):e10105. doi: 10.15252/msb.202010105 (PMC8444284; doi:10.15252/msb.202010105)
Supplement: Supplementary file 6 — Source Data for Figure 5 [file MSB-17-e10105-s004.zip › Figure5A_sourcedata/GSEA_3071/hallmarks_stateI.GseaPreranked.1623742531165/HALLMARK_G2M_CHECKPOINT.html]

Details for gene set HALLMARK\_G2M\_CHECKPOINT[GSEA]

|  || Dataset | state13071 |
| Phenotype | NoPhenotypeAvailable |
| Upregulated in class | na\_neg |
| GeneSet | HALLMARK\_G2M\_CHECKPOINT |
| Enrichment Score (ES) | -0.49112839 |
| Normalized Enrichment Score (NES) | -2.6570082 |
| Nominal p-value | 0.0 |
| FDR q-value | 0.0 |
| FWER p-Value | 0.0 |
Table: GSEA Results Summary

  

Fig 1: Enrichment plot: HALLMARK\_G2M\_CHECKPOINT      
 Profile of the Running ES Score & Positions of GeneSet Members on the Rank Ordered List

  

| PROBE | GENE SYMBOL | GENE\_TITLE | RANK IN GENE LIST | RANK METRIC SCORE | RUNNING ES | CORE ENRICHMENT || 1 | TLE3 |  |  | 126 | 0.968 | -0.0229 | No |
| 2 | TGFB1 |  |  | 165 | 0.884 | -0.0210 | No |
| 3 | NOTCH2 |  |  | 446 | 0.551 | -0.0954 | No |
| 4 | SLC12A2 |  |  | 903 | 0.350 | -0.2247 | No |
| 5 | SLC38A1 |  |  | 1021 | 0.323 | -0.2544 | No |
| 6 | SLC7A5 |  |  | 1084 | 0.310 | -0.2682 | No |
| 7 | SQLE |  |  | 1121 | 0.303 | -0.2743 | No |
| 8 | PML |  |  | 1211 | 0.286 | -0.2963 | No |
| 9 | SLC7A1 |  |  | 1389 | 0.254 | -0.3448 | No |
| 10 | CTCF |  |  | 1450 | -0.253 | -0.3587 | No |
| 11 | SRSF10 |  |  | 1489 | -0.256 | -0.3661 | No |
| 12 | SFPQ |  |  | 1536 | -0.261 | -0.3758 | No |
| 13 | HSPA8 |  |  | 1605 | -0.268 | -0.3919 | No |
| 14 | TTK |  |  | 1620 | -0.270 | -0.3921 | No |
| 15 | STAG1 |  |  | 1624 | -0.270 | -0.3890 | No |
| 16 | BUB3 |  |  | 1687 | -0.278 | -0.4032 | No |
| 17 | RAD54L |  |  | 1702 | -0.280 | -0.4031 | No |
| 18 | KIF2C |  |  | 1735 | -0.284 | -0.4084 | No |
| 19 | CCNB2 |  |  | 1757 | -0.288 | -0.4103 | No |
| 20 | TROAP |  |  | 1821 | -0.296 | -0.4245 | No |
| 21 | CASP8AP2 |  |  | 1857 | -0.300 | -0.4304 | No |
| 22 | KPNA2 |  |  | 1944 | -0.312 | -0.4512 | No |
| 23 | CDC25A |  |  | 1953 | -0.313 | -0.4489 | No |
| 24 | INCENP |  |  | 2018 | -0.320 | -0.4631 | No |
| 25 | DDX39A |  |  | 2043 | -0.324 | -0.4653 | No |
| 26 | CBX1 |  |  | 2096 | -0.334 | -0.4757 | No |
| 27 | CHEK1 |  |  | 2108 | -0.336 | -0.4740 | No |
| 28 | PLK4 |  |  | 2167 | -0.346 | -0.4860 | Yes |
| 29 | RPS6KA5 |  |  | 2173 | -0.347 | -0.4823 | Yes |
| 30 | GSPT1 |  |  | 2177 | -0.348 | -0.4781 | Yes |
| 31 | TNPO2 |  |  | 2180 | -0.348 | -0.4735 | Yes |
| 32 | PLK1 |  |  | 2206 | -0.353 | -0.4757 | Yes |
| 33 | CENPA |  |  | 2220 | -0.357 | -0.4742 | Yes |
| 34 | ILF3 |  |  | 2222 | -0.358 | -0.4692 | Yes |
| 35 | KMT5A |  |  | 2228 | -0.360 | -0.4654 | Yes |
| 36 | SMC1A |  |  | 2240 | -0.361 | -0.4633 | Yes |
| 37 | POLE |  |  | 2252 | -0.364 | -0.4612 | Yes |
| 38 | POLQ |  |  | 2253 | -0.364 | -0.4558 | Yes |
| 39 | CKS1B |  |  | 2332 | -0.380 | -0.4732 | Yes |
| 40 | MCM2 |  |  | 2349 | -0.383 | -0.4722 | Yes |
| 41 | NUP50 |  |  | 2354 | -0.385 | -0.4677 | Yes |
| 42 | DKC1 |  |  | 2365 | -0.386 | -0.4650 | Yes |
| 43 | CCNF |  |  | 2370 | -0.387 | -0.4604 | Yes |
| 44 | STIL |  |  | 2374 | -0.388 | -0.4556 | Yes |
| 45 | PRIM2 |  |  | 2409 | -0.394 | -0.4598 | Yes |
| 46 | BUB1 |  |  | 2416 | -0.396 | -0.4557 | Yes |
| 47 | HNRNPU |  |  | 2434 | -0.398 | -0.4548 | Yes |
| 48 | POLA2 |  |  | 2452 | -0.401 | -0.4539 | Yes |
| 49 | CKS2 |  |  | 2466 | -0.404 | -0.4518 | Yes |
| 50 | AURKA |  |  | 2467 | -0.404 | -0.4458 | Yes |
| 51 | KIF11 |  |  | 2488 | -0.410 | -0.4456 | Yes |
| 52 | RACGAP1 |  |  | 2522 | -0.419 | -0.4492 | Yes |
| 53 | CDC20 |  |  | 2540 | -0.425 | -0.4479 | Yes |
| 54 | CCNA2 |  |  | 2667 | -0.462 | -0.4782 | Yes |
| 55 | KIF22 |  |  | 2683 | -0.468 | -0.4757 | Yes |
| 56 | MCM6 |  |  | 2699 | -0.471 | -0.4732 | Yes |
| 57 | SNRPD1 |  |  | 2701 | -0.471 | -0.4665 | Yes |
| 58 | PRC1 |  |  | 2705 | -0.472 | -0.4604 | Yes |
| 59 | NDC80 |  |  | 2723 | -0.478 | -0.4584 | Yes |
| 60 | EXO1 |  |  | 2728 | -0.479 | -0.4525 | Yes |
| 61 | CENPF |  |  | 2761 | -0.490 | -0.4546 | Yes |
| 62 | BRCA2 |  |  | 2772 | -0.495 | -0.4503 | Yes |
| 63 | SRSF2 |  |  | 2782 | -0.497 | -0.4456 | Yes |
| 64 | DTYMK |  |  | 2798 | -0.501 | -0.4426 | Yes |
| 65 | RBL1 |  |  | 2808 | -0.503 | -0.4378 | Yes |
| 66 | HMGB3 |  |  | 2839 | -0.516 | -0.4390 | Yes |
| 67 | TOP2A |  |  | 2842 | -0.517 | -0.4320 | Yes |
| 68 | RAD21 |  |  | 2866 | -0.527 | -0.4310 | Yes |
| 69 | PTTG1 |  |  | 2895 | -0.540 | -0.4313 | Yes |
| 70 | CDKN2C |  |  | 2903 | -0.544 | -0.4253 | Yes |
| 71 | MT2A |  |  | 2910 | -0.547 | -0.4190 | Yes |
| 72 | TRA2B |  |  | 2933 | -0.563 | -0.4171 | Yes |
| 73 | KIF4A |  |  | 2953 | -0.574 | -0.4143 | Yes |
| 74 | TACC3 |  |  | 2956 | -0.575 | -0.4063 | Yes |
| 75 | KIF23 |  |  | 2961 | -0.579 | -0.3990 | Yes |
| 76 | SMARCC1 |  |  | 2973 | -0.584 | -0.3936 | Yes |
| 77 | SMC2 |  |  | 2984 | -0.592 | -0.3878 | Yes |
| 78 | KNL1 |  |  | 2986 | -0.592 | -0.3793 | Yes |
| 79 | TPX2 |  |  | 3006 | -0.606 | -0.3760 | Yes |
| 80 | TFDP1 |  |  | 3027 | -0.617 | -0.3727 | Yes |
| 81 | AURKB |  |  | 3056 | -0.639 | -0.3716 | Yes |
| 82 | JPT1 |  |  | 3062 | -0.644 | -0.3635 | Yes |
| 83 | CDKN1B |  |  | 3065 | -0.646 | -0.3545 | Yes |
| 84 | CCND1 |  |  | 3072 | -0.655 | -0.3466 | Yes |
| 85 | CDC45 |  |  | 3081 | -0.661 | -0.3392 | Yes |
| 86 | TMPO |  |  | 3088 | -0.666 | -0.3311 | Yes |
| 87 | SAP30 |  |  | 3096 | -0.671 | -0.3233 | Yes |
| 88 | MAD2L1 |  |  | 3099 | -0.675 | -0.3139 | Yes |
| 89 | NASP |  |  | 3106 | -0.682 | -0.3056 | Yes |
| 90 | DBF4 |  |  | 3117 | -0.690 | -0.2983 | Yes |
| 91 | LMNB1 |  |  | 3121 | -0.692 | -0.2890 | Yes |
| 92 | SMC4 |  |  | 3126 | -0.699 | -0.2798 | Yes |
| 93 | MCM5 |  |  | 3132 | -0.706 | -0.2709 | Yes |
| 94 | MEIS1 |  |  | 3135 | -0.711 | -0.2609 | Yes |
| 95 | KIF15 |  |  | 3142 | -0.723 | -0.2520 | Yes |
| 96 | CDK1 |  |  | 3149 | -0.727 | -0.2431 | Yes |
| 97 | HNRNPD |  |  | 3157 | -0.738 | -0.2342 | Yes |
| 98 | MKI67 |  |  | 3158 | -0.738 | -0.2233 | Yes |
| 99 | ORC6 |  |  | 3159 | -0.738 | -0.2124 | Yes |
| 100 | STMN1 |  |  | 3171 | -0.751 | -0.2045 | Yes |
| 101 | MCM3 |  |  | 3182 | -0.761 | -0.1962 | Yes |
| 102 | BARD1 |  |  | 3201 | -0.783 | -0.1900 | Yes |
| 103 | CDC6 |  |  | 3204 | -0.785 | -0.1789 | Yes |
| 104 | BIRC5 |  |  | 3208 | -0.793 | -0.1681 | Yes |
| 105 | FBXO5 |  |  | 3212 | -0.797 | -0.1572 | Yes |
| 106 | UBE2C |  |  | 3225 | -0.812 | -0.1487 | Yes |
| 107 | FOXN3 |  |  | 3234 | -0.824 | -0.1389 | Yes |
| 108 | NUSAP1 |  |  | 3237 | -0.825 | -0.1273 | Yes |
| 109 | HMGN2 |  |  | 3242 | -0.833 | -0.1162 | Yes |
| 110 | ODC1 |  |  | 3257 | -0.860 | -0.1076 | Yes |
| 111 | CHAF1A |  |  | 3270 | -0.882 | -0.0981 | Yes |
| 112 | NSD2 |  |  | 3281 | -0.896 | -0.0878 | Yes |
| 113 | E2F1 |  |  | 3289 | -0.911 | -0.0764 | Yes |
| 114 | CDKN3 |  |  | 3293 | -0.916 | -0.0637 | Yes |
| 115 | HMGA1 |  |  | 3299 | -0.931 | -0.0514 | Yes |
| 116 | MYBL2 |  |  | 3340 | -1.006 | -0.0484 | Yes |
| 117 | EZH2 |  |  | 3342 | -1.015 | -0.0336 | Yes |
| 118 | PBK |  |  | 3354 | -1.046 | -0.0214 | Yes |
| 119 | GINS2 |  |  | 3386 | -1.156 | -0.0135 | Yes |
| 120 | UBE2S |  |  | 3396 | -1.205 | 0.0017 | Yes |
| 121 | MEIS2 |  |  | 3491 | -2.160 | 0.0059 | Yes |
Table: GSEA details [plain text format]

  

Fig 2: HALLMARK\_G2M\_CHECKPOINT: Random ES distribution      
 Gene set null distribution of ES for **HALLMARK\_G2M\_CHECKPOINT**
